# Relationship among psychological distance, social skills, and trait shyness in female university students

**DOI:** 10.3389/fpsyg.2026.1716847

**Published:** 2026-04-29

**Authors:** Noriko Fukutake, Masateru Matsushita, Naoko Yamada

**Affiliations:** Department of Psychology, Faculty of Psychology, Konan Women's University, Kobe, Japan

**Keywords:** female bonding, mother–daughter relationships, psychological distance, social skills, trait shyness

## Abstract

**Introduction:**

Appropriate psychological distance is vital for good relationships. However, the influence of individual factors on this distance remains poorly understood across various social contexts. This study investigated how social skills and trait shyness relate to psychological distance toward different targets (mother, closest friend, and acquaintance) among female university students.

**Methods:**

A cross-sectional survey was conducted with 170 female students (Mean age = 19.3) recruited via convenience sampling. Participants completed the Kikuchi’s Social Skill Scale-18, the Trait Shyness Scale, and a psychological distance scale. Pearson’s correlation and multiple regression analyses examined the predictors of distance for each target.

**Results:**

Psychological distance toward mothers was significantly associated with lower social skills (*r* = −0.27, *p* < 0.001), confirmed in the multiple regression analysis (*β* = −0.35, *p* = 0.002). For closest friends, psychological distance correlated with lower social skills (*r* = −0.18, *p* = 0.023) and higher trait shyness (*r* = 0.15, *p* = 0.049); however, no significant predictors emerged in the regression analysis. Regarding acquaintances, correlations were found with lower social skills (*r* = −0.30, *p* < 0.001) and higher trait shyness (*r* = 0.25, *p* = 0.001), though the regression analysis indicated only social skills remained a significant predictor (*β* = −0.24, *p* = 0.033).

**Discussion:**

The influence of social skills and shyness on psychological distance varies by target. Notably, social skills play a more crucial role than trait shyness in maintaining appropriate psychological distance, even in highly intimate relationships like that with one’s mother.

## Introduction

Maintaining good interpersonal relationships requires establishing an appropriate psychological distance between individuals (Miyama, 2003). This can be observed in psychiatric clinical settings, where therapist–patient relationships are often described using terms implying psychological distance, such as “maintaining distance” or “becoming entangled” (Katsuki, 2009). Previous research suggests that psychological distance is a crucial factor influencing the quality of interpersonal relationships (Sakamoto and Takahashi, 2009).

Psychological distance is the perceived “distance” in the relationship between oneself and others. Previous research offers various definitions. For example, Yamaguchi et al. (1996) described psychological distance in student–teacher relationships as the degree or extent of intimacy, affinity, and closeness between two individuals.

Conversely, Kaneko (1989), after reviewing existing definitions and related concepts, argued that “psychological distance” is more appropriate when comparing the level of psychological connection between oneself and various targets. Kaneko (1989) defined psychological distance as the degree to which an individual feels a strong psychological connection, intimacy, and mutual understanding with another person, considering it a better concept for assessing subjective connections with different relationship targets.

Understanding how interpersonal relationships form requires examining individual communication abilities as a factor influencing psychological distance. Communication fosters mutual understanding but can both facilitate and hinder relationship formation (Kimura et al., 2011). Therefore, this study focuses on social skills and trait shyness as aspects of communication competence.

Social skills are behavioral competencies that promote smooth interpersonal interactions, encouraging positive and avoiding negative responses from others (Kikuchi, 2004). Therefore, social skills are indispensable for establishing, maintaining, and developing relationships with others.

Shyness, as defined by Leary and Atherton (1986), is “a syndrome residing within the individual across specific social situations, characterized by the emotional state of social anxiety and the personal characteristic of interpersonal inhibition.” Shyness can be temporary (state shyness) or relatively stable (trait shyness), with trait shyness manifesting regardless of situation. While state shyness only occurs in specific situations, trait shyness manifests irrespective of context (Aikawa, 1991). To capture these enduring tendencies, this study focuses on trait shyness.

Previous research on psychological distance often examined it in specific contexts of relationships. For example, Kaneko (1989) examined psychological distance in parent–child and peer relationships among adolescent females. Kaneko found no significant difference in psychological distance between adolescent females and their mothers compared to their same-sex friends. Furthermore, female university students tended to have more intimate relationships with their mothers than female high school students. Similarly, Mizumoto (2018) found that daughters exhibit greater intimacy with mothers than fathers, whereas this difference was less pronounced for sons. These studies indicate that women tend to reduce psychological distance with close same-sex individuals and that mother–daughter relationships in early adulthood may foster mutually respectful connections. Previous studies (Kaneko, 1989; Mizumoto, 2018) have showed that women exhibit significant changes in psychological distance from their mothers and same-sex friends during their developmental stages. By focusing on female university students, who clearly manifest these developmental dynamics, we aimed to identify the influence of social skills on psychological distance regulation. Therefore, this study specifically targeted female participants to better capture these interpersonal mechanisms.

However, empirical examinations on how psychological distance is shaped or maintained by individual characteristics are limited. We hypothesized that individuals with high social skills would form more intimate relationships through smoother interactions, whereas those with high trait shyness would avoid interactions, leading to greater psychological distance.

Accordingly, this study aims to empirically clarify the relationships among psychological distance, social skills, and trait shyness. This investigation is expected to form an understanding of the factors that shape and maintain interpersonal relationships. Therefore, this study examines these relationships in female university students.

## Methods

### Participants and procedure

This study employed a cross-sectional survey design. Participants were recruited using a convenience sampling technique from students enrolled in several introductory psychology courses at a university in Japan. Before the lectures, the researchers distributed a document explaining the study’s purpose, the voluntary nature of participation, and the assurance of anonymity. Informed consent was obtained from all participants prior to data collection. The survey was conducted anonymously, and no personally identifiable information was collected. Participants were assured that their participation would have no effect on their academic grades. The inclusion criterion was being a female undergraduate student. Exclusion criteria included insufficient comprehension of Japanese or excessive missing values. Ultimately, no participants were excluded, and all 170 recruited students (Mean age = 19.3 years, SD = 1.21) were included. Data collection was conducted between June 2024 and August 2025. Sociodemographic information, including age and academic year, was utilized in the analysis.

### Measurement

This study measured psychological distance, social skills, and trait shyness using the following questionnaires.

#### Psychological distance scale

Psychological distance was measured using Kaneko’s Psychological Distance Scale (Kaneko, 1989). Participants rated their perceived distance from three targets: “a female acquaintance,” “closest friend at your university,” and “mother.” This scale comprises 10 items, each rated on a 5-point Likert scale, ranging from 1 (not at all applicable) to 5 (highly applicable), with 3 indicating ‘neither applicable nor inapplicable.’ The total score ranges from 10 to 50, with higher scores indicating greater psychological distance (Kaneko, 1989). A pilot test identified an ambiguously worded item, which was revised for clarity.

#### Kikuchi’s social skill scale

Social skills were assessed using the Kikuchi’s (2007) 18-item Social Skills Scale (KiSS-18). The scale consists of 18 items, each rated on a 5-point Likert scale: 1 (never), 2 (rarely), 3 (sometimes), 4 (usually), and 5 (always). The total score ranges from 18 to 90, with higher scores indicating higher levels of social skills.

#### Trait shyness scale

Trait shyness was measured using Aikawa’s (1991) Japanese version of the 16-item Trait Shyness Scale. Each item was rated on a 5-point Likert scale: 1 (not at all applicable), 2 (not very applicable), 3 (neither applicable nor inapplicable), 4 (somewhat applicable), and 5 (highly applicable). The total score ranges from 16 to 80, with higher scores indicating higher levels of trait shyness, reflecting social anxiety and interpersonal inhibition (Leary and Atherton, 1986). A pilot test identified one unclear item, which was reworded for improved clarity and comprehensibility.

### Statistical analyses

This study utilized Pearson’s correlation and multiple regression analyses to examine the relationships among psychological distance, social skills, and trait shyness. Significance was set at *p* < 0.05. All analyses were conducted using SPSS Windows software version 20.0.

### Ethical considerations

This study was approved by the Konan Women’s University Graduate School Ethics Review Board (approval number: 2025021).

## Results

Table 1 presents the sociodemographic characteristics of the participants and the descriptive statistics for all primary variables. Among the 170 participants, 84 were first-year students (49.4%), 15 were second-year students (8.8%), 51 were third-year students (30.0%), and 20 were fourth-year students (11.8%). The mean age of the participants was 19.3 years (SD = 1.21).

**Table 1:** Means and standard errors for psychological distance, social skills, and trait shyness.

Variable	First-year students (*n* = 84, 49.4%)		Second-year students (*n* = 15, 8.8%)		Third-year students (*n* = 51, 30.0%)		Fourth-year students (*n* = 20, 11.8%)		Total (*n* = 170, 100%)	
	Mean	SE	Mean	SE	Mean	SE	Mean	SE	Mean	SE
Psychological distance with mother	21.6	0.90	23.7	2.12	19.5	1.15	22.2	1.84	21.7	0.79
Psychological distance with close friend	22.1	0.88	23.4	2.07	20.7	1.12	19.6	1.80	21.5	0.77
Psychological distance with an acquaintance	36.3	0.78	35.3	1.85	35.2	1.00	33.1	1.60	35.0	0.69
Social skills	53.0	1.44	49.0	3.40	56.1	1.84	55.2	2.94	53.3	1.27
Trait shyness	53.5	1.64	58.3	3.88	55.0	2.10	47.7	3.36	53.6	1.45

SE expresses standard error.

Prior to the main analysis, the normality of the data distribution was examined, and all variables were within the acceptable range (Table 1). We conducted correlation analyses among the psychological distance scale scores (for mother, closest friend at university, and female acquaintance), KiSS-18 scores, and trait shyness scale scores (Table 2). Scores for psychological distance with one’s mother were positively correlated with those for the closest friend (*r* = 0.175, *p* = 0.023) and negatively correlated with KiSS-18 scores (*r* = −0.267, *p* < 0.001). For the closest friends, distance scores correlated negatively with KiSS-18 scores (*r* = −0.175, *p* = 0.023) and positively with trait shyness scores (*r* = 0.151, *p* = 0.049). For female acquaintances, distance negatively correlated with KiSS-18 scores (*r* = −0.296, *p* < 0.001) and positively with trait shyness scores (*r* = 0.254, *p* = 0.001). KiSS-18 scores were also strongly negatively correlated with trait shyness scores (*r* = −0.750, *p* < 0.001).

**Table 2:** Correlation analysis of scale scores.

Variable	Psychological distance with mother		Psychological distance with close friend		Psychological distance with an acquaintance		Social skills	
	*r*	*p*	*r*	*p*	*r*	*p*	*r*	*p*
Psychological distance with mother	-							
Psychological distance with close friend	0.175	0.023*	-					
Psychological distance with an acquaintance	0.098	0.202	0.129	0.094	-			
Social skills	−0.267	< 0.001***	−0.175	0.023*	−0.296	< 0.001***	-	
Trait shyness	0.149	0.052	0.151	0.049*	0.254	0.001***	−0.75	< 0.001***

***p < 0.001, *p < 0.05.

We conducted multiple regression analyses (forced entry method) for each relationship target (mother, closest friend at university, female acquaintance) with psychological distance scores as dependent variables and KiSS-18 and trait shyness scores as independent variables (Table 3). For mothers, the model was significant (*F*(2, 167) = 6.98, *p* = 0.001), accounting for 7.7% of the variance. KiSS-18 scores negatively associated with psychological distance (*β* = −0.35, *p* = 0.002), whereas trait shyness was not (*β* = −0.12, *p* = 0.303). Neither KiSS-18 scores (*β* = −0.14, *p* = 0.222) nor trait shyness scores (*β* = 0.05, *p* = 0.696) showed a significant association for closest friends.

**Table 3:** Associations of psychological distance with mother, close friend, and acquaintance with social skills and shyness.

Variable	Psychological distance with mother			Psychological distance with close friend			Psychological distance with an acquaintance		
	*β*	*t*	*p*	*β*	*t*	*p*	*β*	*t*	*p*
Social skills	−0.35	−3.15	0.002**	−0.14	−1.23	0.222	−0.24	−2.15	0.033*
Trait shyness	−0.12	−1.03	0.303	0.05	0.39	0.696	0.07	0.66	0.508
Adjusted *R*^2^	0.066			0.020			0.079		

**p < 0.01, *p < 0.05.

For female acquaintances, the model was significant (*F*(2, 167) = 8.237, *p* < 0.001), explaining 9.0% of the variance. KiSS-18 scores were negatively associated with psychological distance (*β* = −0.24, *p* = 0.033), while trait shyness was not (*β* = 0.07, *p* = 0.508). In all models, Variance Inflation Factor values were <10, indicating no multicollinearity.

## Discussion

This study examined how psychological distance relates to social skills and trait shyness among 170 female university students. The correlation analysis revealed several notable patterns. Closer psychological distance with one’s mother correlated with closer psychological distance with one’s closest friend. Additionally, closer psychological distance (for most targets) was associated with lower social skills and slightly higher trait shyness. Finally, higher social skills were correlated with lower trait shyness.

These findings suggest that closer mother–daughter relationships may be associated with stronger friendships. The negative correlation between social skills and trait shyness indicates that individuals more adaptable in social situations tend to experience less interpersonal anxiety. The consistent negative correlation between social skills and psychological distance and the positive correlation between trait shyness and psychological distance suggest a nuanced pattern. Individuals may form intimate relationships with specific people yet struggle to expand their social networks. This implies that intimacy and sociability may represent distinct aspects of interpersonal functioning. Regression results showed that social skills were negatively associated with psychological distance for mothers and female acquaintances, whereas trait shyness was not. For the closest friends, neither variable showed a significant association. These results align with research showing that higher social skills facilitate intimate relationships through smoother interactions (Kikuchi, 2007). Such skills may also lead individuals to perceive others as more intimate, resulting in shorter reported closer psychological distances (Kikuchi, 2007).

However, the absence of this association for closest friends may reflect the unique nature of such friendships. In such relationships, mutual understanding and trust are often well-established through shared experiences and time spent together over time (Kimura et al., 2011). In these contexts, social skills may be less essential for maintaining the relationship. In highly intimate relationships, a sense of “comfort” and “security”—where interaction occurs without reservation—may be prioritized over social skills (Kimura et al., 2011). Our findings align with recent trends in interpersonal relationship research. Specifically, the result that social skills and trait shyness had limited influence on psychological distance in close friendships is consistent with the findings of Baker and McNulty (2010). They demonstrated that in established, intimate relationships, even shy individuals experience reduced evaluative anxiety, leading to increased self-disclosure and a diminished impact of personality traits on relationship maintenance. In contrast, for acquaintances where mutual understanding is not yet fully formed, social skills are more likely to influence the regulation of psychological distance. These results suggest that trait shyness does not unilaterally increase psychological distance; rather, its impact is moderated by the closeness of the relationship. Thus, our study supports the recent perspective that the manifestation of shyness is highly context dependent. This could explain why the social skills measured in this study did not directly influence psychological distance in these specific close friendships.

Several factors may explain the lack of association between trait shyness and psychological distance across all relationship types. Even highly shy individuals may feel less evaluative anxiety in established relationships, allowing for more self-disclosure and limiting effects on psychological distance (Baker and McNulty, 2010). For acquaintances, where psychological distance is typically greater, trait shyness may have limited influence. Additionally, while trait shyness is defined as “a syndrome residing within the individual across specific social situations, characterized by the emotional state of social anxiety and the personal characteristic of interpersonal inhibition” (Leary and Atherton, 1986), our psychological distance scale focuses on the “state” or “feeling” of a relationship. This difference in focus might be relevant; the behavioral tendencies indicated by trait shyness may not directly translate to, or may have limited impact on, the subjective sense of “psychological connection.” This point suggests that further investigation is needed into how the various facets of trait shyness, including its behavioral aspects, influence psychological distance. These findings indicate that trait shyness may not function in a straightforward, unidirectional manner, and that even individuals with high trait shyness may not necessarily experience greater psychological distance.

Our finding that psychological distance is closest with mothers aligns with Kaneko’s (1989) observation that female university students tend to have more intimate mother–daughter relationships than female high school students. The finding that psychological distance was shortest with mothers is consistent with recent research. For instance, Morikawa (2025) suggested that a stable psychological distance with one’s biological mother is associated with higher resilience. Furthermore, Mizumoto (2019) reported that modern female emerging adults achieve psychological independence while maintaining a form of ‘dependent intimacy’ with their mothers. These findings suggest that the mother-daughter relationship plays a foundational role in the formation of interpersonal relationships among women. This study contributes to understanding psychological distance in interpersonal relationships and how social skills affect these connections. In particular, the finding that social skills reduce psychological distance in relationships with mothers and acquaintances offers new insights into relationship formation mechanisms. These results could guide psychological support settings by identifying skills individuals with interpersonal relationship difficulties may cultivate to foster healthier connections. They also reinforce the importance of social skills training in educational and counseling environments.

A strength of this study is its multifaceted examination of how social skills and trait shyness influence psychological distance across relationship targets. This approach suggests that, depending on interpersonal context, individual traits may affect psychological distance.

This study has some limitations that suggest directions for future research. First, since this study was cross-sectional, longitudinal research is needed to see how psychological distance changes over time (Mizumoto, 2019). Second, we used subjective ratings to measure psychological distance. Future studies should also use behavioral or physiological data for a more complete evaluation. Third, mother-daughter intimacy may be unique to Japanese culture. Comparing different cultures would help identify universal patterns. Finally, future research should test if social skills training helps reduce psychological distance. This could be especially useful for building new relationships in educational or clinical settings.

This study clarifies the role of social skills and trait shyness in psychological distance within interpersonal relationships. A key finding is that social skills reduce psychological distance, particularly with mothers and acquaintances, whereas trait shyness showed no direct influence. This novel insight provides valuable information for understanding relationship formation mechanisms. They also highlight which skills are important for establishing and maintaining relationships. This information may guide future theoretical work and inform practical interventions in interpersonal relationship research.

## Data Availability

The original contributions presented in the study are included in the article, further inquiries can be directed to the corresponding author.
